# Evaluation of residual cognition in patients with disorders of consciousness based on functional near-infrared spectroscopy

**DOI:** 10.1117/1.NPh.10.2.025003

**Published:** 2023-04-12

**Authors:** Juanning Si, Yi Yang, Long Xu, Tianshuai Xu, Hao Liu, Yujin Zhang, Rixing Jing, Jinglian Li, Dongdong Wang, Sijin Wu, Jianghong He

**Affiliations:** a Beijing Information Science and Technology University, School of Instrumentation Science and Opto-Electronics Engineering, Beijing, China; b Beijing Tiantan Hospital, Capital Medical University, Department of Neurosurgery, Beijing, China; c Chinese Academy of Sciences, Institute of Automation, Brainnetome Center, Beijing, China; d Chinese Academy of Sciences, Institute of Automation, National Laboratory of Pattern Recognition, Beijing, China; e Sanhe Yanjiao Fuhe First Hospital, Department of Neurosurgery, Langfang, China

**Keywords:** disorders of consciousness, functional near-infrared spectroscopy, residual awareness, motor imagery, hemodynamic response

## Abstract

**Significance:**

Accurate evaluation of consciousness in patients with prolonged disorders of consciousness (DOC) is critical for designing therapeutic plans, determining rehabilitative services, and predicting prognosis. Effective ways for detecting consciousness in patients with DOC are still needed.

**Aim:**

Evaluation of the residual awareness in patients with DOC and investigation of the spatiotemporal differences in the hemodynamic responses between the minimally conscious state (MCS) and the unresponsive wakefulness syndrome (UWS) groups using active command-driven motor imagery (MI) tasks.

**Approach:**

In this study, functional near-infrared spectroscopy (fNIRS) was used to measure the changes of hemodynamic responses in 19 patients with DOC (9 MCS and 10 UWS) using active command-driven MI tasks. The characteristics of the hemodynamic responses were extracted to compare the differences between the MCS and UWS groups. Moreover, the correlations between the hemodynamic responses and the clinical behavioral evaluations were also studied.

**Results:**

The results showed significant differences in the spatiotemporal distribution of the hemodynamic responses between the MCS and UWS groups. For the patients with MCS, significant increases in task-evoked hemodynamic responses occurred during the “YES” questions of the command-driven MI tasks. Importantly, these changes were significantly correlated with their coma-recovery scale-revised (CRS-R) scores. However, for the patients with UWS, no significant changes of the hemodynamic responses were found. Additionally, the results did not show any statistical correlation between the hemodynamic responses and their CRS-R scores.

**Conclusions:**

The fNIRS-based command-driven MI tasks can be used as a promising tool for detecting residual awareness in patients with DOC. We hope that the findings and the active paradigm used in this study will provide useful insights into the diagnosis, therapy, and prognosis of this challenging patient population.

## 1 Introduction

Accurate diagnosis of the level of consciousness in patients with prolonged disorders of consciousness (DOC) is critical for designing therapeutic plans, determining rehabilitative services, and predicting prognosis. The DOC^1^^–^^4^ mainly include coma (unwakefulness and reflex behavior only), unresponsive wakefulness syndrome (UWS), and minimally conscious state (MCS). Patients in UWS^5^ are clinically awake but unaware of themselves and the surrounding environment. Unlike UWS, patients in MCS^6^ present minimal, inconsistent, but reproducible purposeful evidence of awareness. The clinical evaluation of awareness and diagnosis of these two groups of patients is challenging. At present, subjective bedside behavioral assessments are still regarded as the clinical reference standard for evaluating the awareness of patients with DOC.^7^ However, it has been reported that the misdiagnosis rate was ∼40%.^3^ In fact, accurate evaluation of brain function and detection of residual awareness in patients with DOC is complicated, time-consuming, and challenging due to the cognitive, motor, and sensory impairments of the patients, as well as to other factors, such as the examiner and environmental conditions.

An objective way to evaluate the level of consciousness for patients with DOC is using functional neuroimaging technologies. In the past few years, a variety of neuroimaging and electrophysiological studies have provided valuable insights into evaluating awareness, exploring structural and functional characteristics, monitoring the recovery of consciousness, and elucidating the underlying mechanisms of consciousness in patients with DOC.^1^^,^^3^^,^^4^^,^^8^ In 2006, an functional magnetic resonance imaging (fMRI) study was proposed by Owen et al., they instructed a patient who met the clinical criteria of UWS to perform tennis-playing imagery to respond “yes” and to perform spatial navigation imagery to respond “no” while in an fMRI scanner.^9^ The fMRI results showed that the brain activity of the patient was similar to that of healthy controls under the same tasks, indicating residual cognition. In a follow-up study,^10^ fMRI was used to assess the willful brain activities of a group of DOC patients during mental-imagery tasks. The results showed that ∼17% of patients who behaviorally appeared nonresponsive, in fact, had covert awareness and could modulate their brain activities to communicate in response to simple yes-or-no questions. Emerging findings from neuroimaging and electrophysiological studies indicate that about 20% of patients who seem unresponsive on behavioral examination, can in fact, present covert consciousness. This discrepancy is known as cognitive motor dissociation (CMD).^4^^,^^11^ That is, these patients are actually aware even though they are behaviorally nonresponsive, but clear signs of awareness can be observed using electrophysiological and neuroimaging technologies that do not depend on the patient’s ability to produce external responses. Recently, the 2020 European Academy of Neurology guideline recommends that task-based neuroimaging studies should be used for the accurate detection of consciousness for patients with DOC “whenever feasible.”^12^ Although the studies based on fMRI have provide valuable information in the field of DOC, the relatively large-scale equipment requirements, expense, and strict subject constraints preclude its application for patients with metal implants and limit its use for longitudinal bed-side monitoring or for patients in critical condition in intensive care units.

Functional near-infrared spectroscopy (fNIRS)^13^ is a non-invasive optical neuroimaging technology that is developing rapidly in the fields of brain-computer interface (BCI), schizophrenia research, neurorehabilitation, neonatal care, etc.^7^^,^^14^^–^^16^ Compared to fMRI, fNIRS is more tolerant of motion artifacts and metal implants. It provides more comprehensive information to better characterize the hemodynamics with increased ecological validity by detecting the changes of oxygenated (HbO), deoxygenated (HbR), and total (HbT) hemoglobin concentrations simultaneously. In addition, fNIRS is superior to EEG in its ability to localize and segment brain areas at a higher spatial resolution. fNIRS is also more cost-effective, portable, and can be used for bedside monitoring, allowing it to be suitable for longitudinal and repeated monitoring of the hemodynamics of patients with DOC. However, studies using fNIRS to investigate the functional activity and evaluate residual awareness of patients with DOC are still limited but increasing.^3^^,^^7^^,^^17^^–^^20^ In 2013, Molteni et al.^18^ first used fNIRS for the bedside assessment of residual awareness in patients with MCS. Kempny et al.^17^ evaluated the brain activity of patients with DOC by using an fNIRS-based motor imagery (MI) task; the results showed that, compared with UWS patients, the hemodynamic responses of the MCS patients were more similar to those of the healthy controls. Abdalmalak and his colleagues^7^^,^^19^ also applied a fNIRS-based MI task to communicate with patients with DOC and later suggested that fNIRS-based BCI could be a promising tool for improving the quality of life in patients with DOC.^7^ Mental arithmetic tasks have also been utilized to evaluate residual awareness in patients with MCS.^21^ The same task-evoked hemodynamic responses typically found in healthy people during a mental arithmetic task were also obtained under a specific set of conditions in a single MCS patient.^21^ Emerging evidence indicates that fNIRS can be used as an important auxiliary to fMRI to provide new insights into the field of DOC. Although these findings are promising, further explorations and improvements are essential because the studies based on fNIRS are fragmented and limited at present. In addition, the differences in hemodynamics between patients with MCS and UWS and the underlying mechanisms that cause them to differ still need to be elucidated. In our previous study,^22^ fNIRS was used to detect residual awareness in DOC patients during a MI task. A support vector machine was used to classify the yes-or-no responses. The study further confirmed the feasibility and effectiveness of fNIRS for investigating the functional activity of patients with MCS using active command-driven MI tasks. However, because of the limited sample size, the previous results require further experimental investigation and clinical confirmation.

In view of the above-mentioned issues, the research purposes of the current study were as follows. One of the purposes was to further evaluate the reliability of the fNIRS-based active command-driven MI paradigm in detecting the residual awareness of patients with DOC. The second purpose was to explore the differences in the spatiotemporal characteristics of the hemodynamic responses between MCS and UWS patients. The third purpose was to confirm the ability of fNIRS-based BCI to establish basic communication for this challenging patient population.

## 2 Materials and Methods

### 2.1 Subjects

Nineteen DOC patients (15 males and 4 females and 14 to 66 years) were recruited from the Department of Neurosurgery, Beijing Tiantan Hospital and the Department of Neurosurgery, Sanhe Yanjiao Fuhe First Hospital. In the current study, the inclusion criteria were as follows, (1) aged from 10 to 68; (2) diagnosed as UWS or MCS using the coma-recovery scale-revised (CRS-R) scale; (3) etiology of traumatic brain injury (TBI), stroke, or anoxia, with a duration of more than 28 days; and (4) able to obtain informed consent from the caregivers. The exclusion criteria were (1) history of epilepsy or psychiatric or neurological disorders, (2) long-term use of sedative or antiepileptic drugs, (3) uncontrollable infections or other serious medical diseases, (4) severe aphasia or impaired cognition, or (5) inability to obtain informed consent. In this study, written informed consent for each subject was obtained from the patient’s legal guardians. Trained clinicians used the CRS-R scale to evaluate the consciousness levels of the enrolled patients with DOC. The CRS-R is a well established and widely used tool for the behavioral assessment of patients with DOC.^23^ The CRS-R consists of six subscales designed to evaluate auditory, visual, motor, oromotor, communication, and arousal functions, which are summed together to yield a total score with a possible range of 0 to 23.^24^ In this study, the consciousness level of each patient was assessed at two different stages. Specifically, CRS-R (t1) refers to the first stage before the fNIRS recordings; CRS-R (t2) refers to the second stage, which occurred 6 months after the fNIRS recordings. The experimental protocol was approved by the ethics committees of the hospitals. The clinical features of the patients with DOC are shown in Table 1.

**Table 1:** Clinical features of patients with DOC.

No	Age/gender	Diagnosis	Duration of DOC (days)	Etiology	CRS-R (t1)	CRS-R (t2)
1	56/M	MCS	197	TBI	8(113102)	10(214102)
2	35/M	MCS	73	Stroke	10(133102)	20(456113)
3	53/M	MCS	63	TBI	11(233102)	11(233102)
4	46/M	UWS	57	Stroke	7(112102)	7(112102)
5	66/M	MCS	219	Stroke	10(133102)	13(334102)
6	21/M	UWS	129	TBI	5(101102)	7(112102)
7	46/M	UWS	164	TBI	7(211102)	21(455223)
8	66/M	UWS	705	Stroke	3(000102)	3(000102)
9	33/M	MCS	128	Stroke	15(450123)	15(450123)
10	65/M	UWS	238	Anoxia	7(112102)	7(112102)
11	42/M	UWS	133	Stroke	6(111102)	12(333102)
12	49/F	MCS	4380	Stroke	18(445122)	18(445122)
13	14/F	MCS	71	TBI	13(233113)	23(456323)
14	53/F	MCS	105	TBI	11(232103)	12(233103)
15	49/F	MCS	264	Anoxia	11(232103)	
16	51/M	UWS	91	Stroke	7(112102)	11(233102)
17	49/M	UWS	201	Stroke	7(122101)	13(233203)
18	41/M	UWS	504	Stroke	8(122102)	11(233102)
19	40/M	UWS	505	Stroke	6(112101)	6(112101)

UWS, unresponsive wakefulness syndrome; MCS, minimally conscious state; CRS-R, coma recovery scale-revised; and TBI, traumatic brain injury.

### 2.2 Study Design

**Fig. 1:**
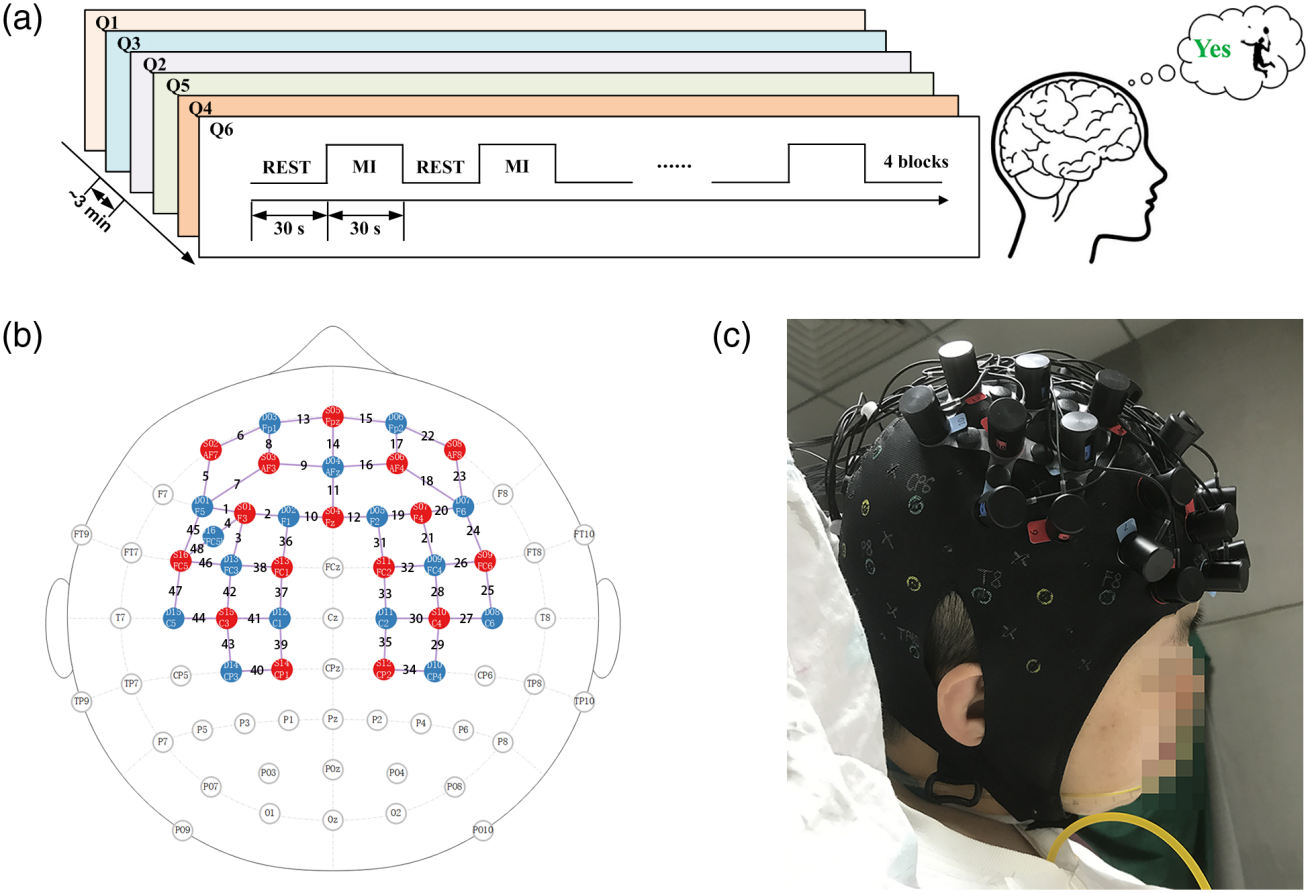
Illustration of the experimental configuration. (a) The experimental paradigm for the command-driven MI tasks. (b) Diagram of the arrangement of the optodes. Specifically, we used 16 sources (red circles) and 16 detectors (blue circles), for a total of 48 optical channels. (c) Photograph of the experimental setup.

In this study, the experimental paradigm was designed based on the well-established tennis-playing MI tasks proposed by Owen in 2006.^9^ Inspired by the promising results proposed by Owen et al, several studies have been conducted to use MI tasks for detection of residual consciousness in patients with DOC^17^^,^^19^^,^^25^^–^^27^ in the past few years. Our previous pilot study^22^ was also based on the tennis-playing MI paradigm. To further confirm the findings of our previous study and to investigate the spatiotemporal characteristics between UWS and MCS groups, the improved experimental paradigm was conducted in the current study. In this study, the patients with DOC were required to perform block-designed command-driven MI tasks. Specifically, the entire experimental paradigm consisted of an initial resting period (10 min) followed by six different question sessions. (1) This question was designed to see whether the subject could understand the experimental paradigm (Q1). (2) This confirmed his/her correct family name (“YES” question, Q2). (3) This asked whether he/she has children or siblings (“YES” question, Q3). (4) This one asked whether this was his/her occupation, which was given incorrectly (“NO” question, Q4). (5) This asked whether there are four seasons in a year (“YES” question, Q5). (6) This one asked whether this was his/her family name, which was given incorrectly (“NO” question, Q6). In view of the familiarity and sport habits of Chinese people, badminton-playing MI tasks was used. Specifically, for each question, if the answer was “YES,” the subject was told to perform first person perspective kinesthetic^28^ badminton-playing MI task by waving his/ her arms continuously to hit a shuttlecock throughout the response period. If the answer was “NO,” then the subject was requested to remain relaxed. Each question session consisted of a baseline period (30 s) followed by four blocks; each block comprised of a response period (30 s) and a recovery period (30 s). To minimize fatigue and to control the overall duration of the experiment, the subject was allowed a 5-min break after each session. Because the auditory pathway is a relatively well-preserved information input channel in patients with DOC,^29^ each question was presented pseudo-randomly using auditory commands (“start answering” and “stay relaxed”). The experimental configuration is shown in Fig. 1.

### 2.3 Data Acquisition

The hemodynamic data were acquired using the NIRScout (NIRx Inc.) system. The system utilizes two wavelengths (760 and 850 nm) to discriminate respectively between the HbR and HbO concentrations in a tissue. In current study, the fNIRS optodes were arranged over the motor-related cortex according to the international 10 to 20 system. Specifically, 16 sources and 16 detectors were placed over the prefrontal cortex (PFC), primary motor cortex (MC), and primary sensorimotor cortex (SC) to yield a total of 48 optical channels, as illustrated in Fig. 1(b). The distance between the source and detector pairs was 30 mm. The sampling rate of the fNIRS system was 10.2 Hz.

### 2.4 Data Analysis

The fNIRS data were analyzed using HOMER 2 and MATLAB 2019a (MathWorks Inc., Natick, Massachusetts, United States). The raw light intensity data were converted into relative changes in oxygenated (HbO) and deoxygenated (HbR) hemoglobin data using the modified Beer-Lambert law.^30^^,^^31^ Specifically, the signal-to-noise ratio (SNR) of raw data of each optical channel was calculated using coefficient of variation (CV=σ/μ).^32^ Where, μ and σ indicate the mean value and the standard deviation of the signal, respectively. In this study, the optical channels with ${\mathrm{CV}}_{\text{channel}}>15\%$ were discarded, and of the remaining optical channels with ${\mathrm{CV}}_{\text{trial}}<5\%$ were used for further analysis. After that, the raw light intensity data were transformed into optical density. The corresponding molar extinction coefficients for HbO and HbR were $\varepsilon_{760\;\mathrm{nm}}=[1486.5865,3843.707]\;{\mathrm{cm}}^{-1}/M$ and $\varepsilon_{850\;\mathrm{nm}}=[2526.391,1798.643]\;{\mathrm{cm}}^{-1}/M$. Then the principal component analysis algorithm^33^^,^^34^ was used for removal extracerebral noises. After that, the hemoglobin data were bandpass-filtered (0.01 to 0.1 Hz) to reject task-unrelated noise, such as heartbeat, breathing cycles, and blood pressure oscillations. It has been reported that the differential pathlength factor (DPF) is related to several factors, such as: wavelength, age of subject, etc. Considering the relatively large difference in the age of the subjects in this study, the DPF value for each subject was calculated based on the method proposed by Scholkmann and Wolf.^35^ Next, the data were segmented into epochs from 5 s before and 40 s after the onset of the response period. Epochs with large motion artifacts were rejected. After a baseline correction (the data during −5 to 0 s was calculated as the baseline), the block-averaged hemodynamic responses were calculated. A typical cerebral activation is characterized by a significant increase in HbO concentration companied by a small decrease in HbR concentration.^17^^,^^20^ In this study, the optical channel with the maximum functional brain activation in each brain area was used for further analysis. Specifically, FC_L (channels 1, 2, 5, 6, 7, 8); FC_R (channels 17, 18, 19, 20, 22, 23); FC_M (channels 9, 10, 11, 12, 13, 14, 15, 16); MC_L (channels 37, 38, 42, 46, 47); MC_R (channels 25, 26, 28, 32, 33); SC_L (channels 39, 40, 41, 43, 44); and SC_R (channels 27, 29, 30, 34, 35), as shown in Fig. 1. For the quantitative analysis, considering that the mean and the slope values have been commonly used as characteristics in the field of fNIRS;^36^ therefore, in this study, the mean and slope values of the hemodynamic responses during the command-driven MI tasks were extracted to investigate the differences between the MCS and UWS groups. Specifically, the mean values of the hemodynamic responses were extracted from 5 to 25 s after the onset of the response period. The slope values were calculated from 2 to 7 s after the onset of the response period. Four patients, numbers 6, 13, 14, and 19 in the Table 1, were rejected from further analysis because of extremely large motion artifacts or large coefficient of variation (CV) values throughout the experiment. For the remaining fNIRS data, the total trial rejection rate was 18.8%.

In this study, two-sample t-tests and one-way analysis of variances (ANOVAs) were conducted to characterize the differences between the different conditions. To correct for multiple comparisons, the false discovery rate method was utilized.^37^ Additionally, the Pearson correlations between the hemodynamic responses and the CRS-R scores were calculated to explore the possible relationships between the hemodynamic responses and the patients’ behavioral evaluations. The differences were accepted as significant when p<.05. The results are provided as means ± standard error (SE), unless otherwise mentioned.

## 3 Results

### 3.1 Hemodynamic Responses During the Command-Driven Motor Imagery Tasks

**Fig. 2:**
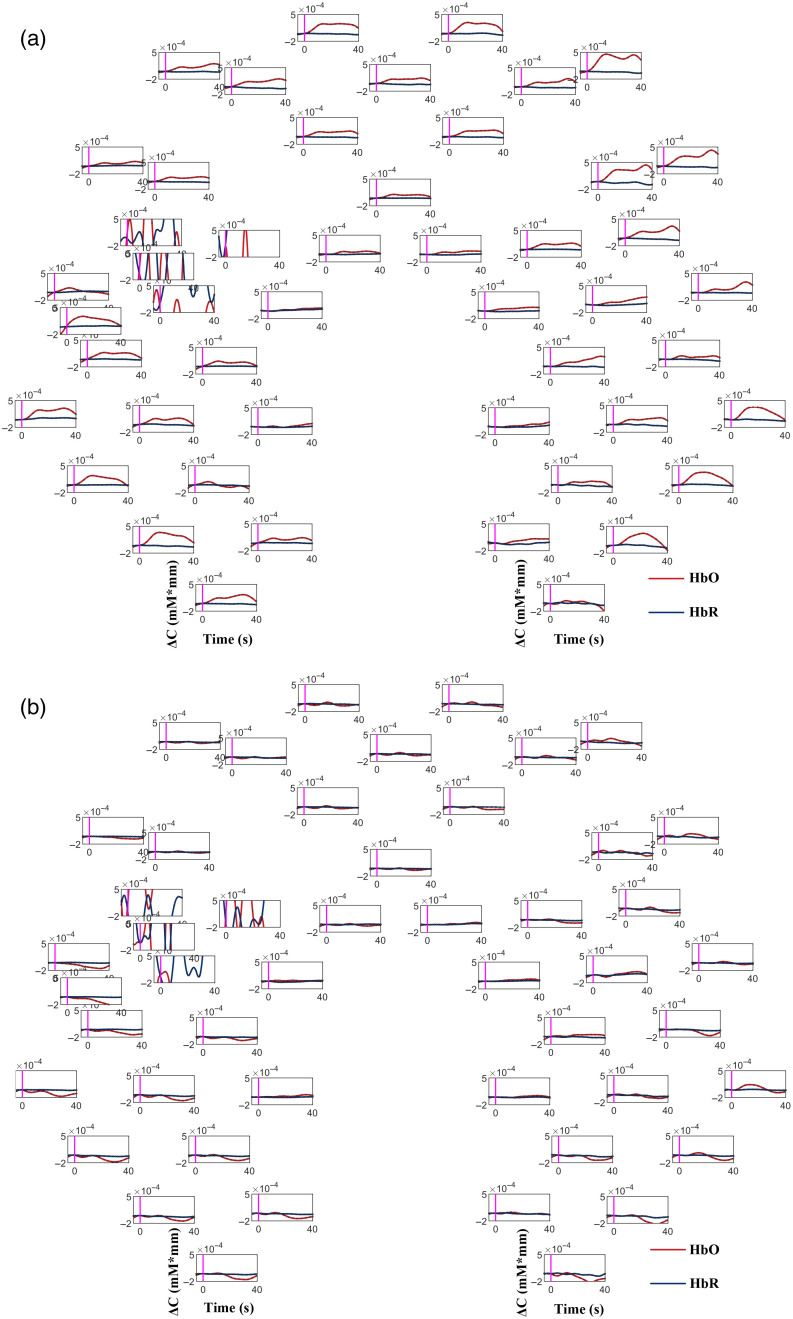
Time courses of the changes in HbO and HbR concentrations during the command-driven MI tasks for an MCS patient (patient No. 9) for one “YES” question (a) and one “NO” question (b). The red and blue lines indicate the HbO and HbR concentrations, respectively. The vertical magenta lines represent the onset of the response period. The positions of each graph reflect the relative positions of the optical channels [see Fig. 1(b)].

**Fig. 3:**
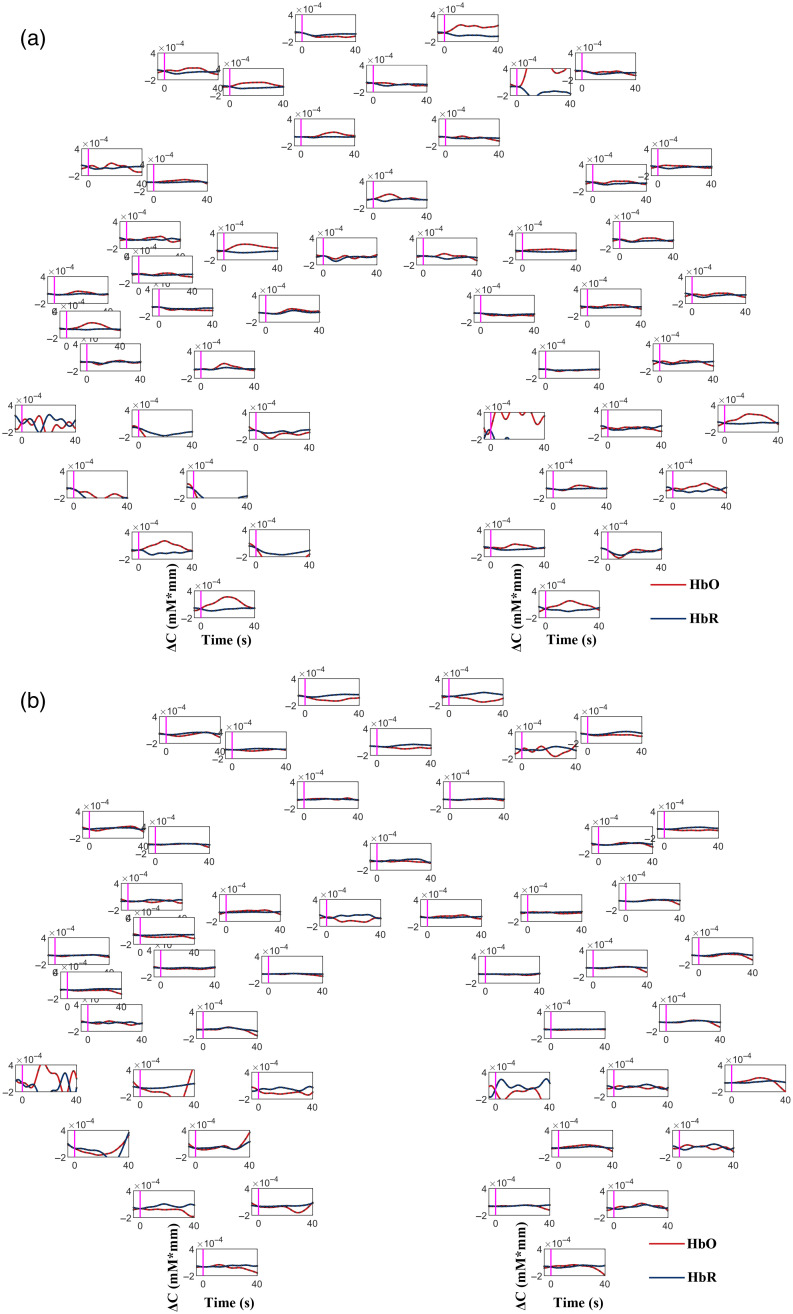
Time courses of the changes in HbO and HbR concentrations during the command-driven MI tasks for a UWS patient (patient No. 7) for one “YES” question (a) and one “NO” question (b). The red and blue lines indicate the HbO and HbR concentrations, respectively. The vertical magenta lines represent the onset of the response period. The positions of each graph reflect the relative positions of the optical channels [see Fig. 1(b)].

The time courses of the changes in hemodynamic responses during the command-driven MI task for typical patients are shown in Figs. 2 and 3. As illustrated in Figs. 2 and 3, the waveforms of the changes of the hemodynamic responses of the MCS patients (patient No. 9) and the UWS (No. 7) showed similar distributions to the typical hemodynamics (i.e., increase in HbO concentrations and decrease in HbR concentrations).

As shown in Fig. 2, the hemodynamic responses for the “YES” and “NO” questions for the MCS patient (patient No. 9) were noticeably different from each other. Specifically, for the “YES” question (Q2: confirm his correct family name), the HbO concentrations over the bilateral primary MC, bilateral primary SC, and bilateral PFC increased significantly, while the HbR concentrations decreased correspondingly after the onset of the task, but the changes in the HbO concentrations were more apparent than those in the HbR concentrations. When the MI tasks were finished, the HbO and HbR concentrations gradually returned to the baseline level. For the “NO” question (Q6: respond that this was not his correct family name), no significant changes in the HbO and HbR concentrations were found consistently over the primary MC, primary SC, and PFC throughout the task periods. Interestingly, as illustrated in Fig. 3, the HbO concentrations of the UWS patient (patient No. 7) during the “YES” question increased noticeably in many channels over the primary motor (MC), primary SC, and prefrontal (FC) cortices. There were no significant changes in the HbO and HbR concentrations for the “NO” question.

**Fig. 4:**
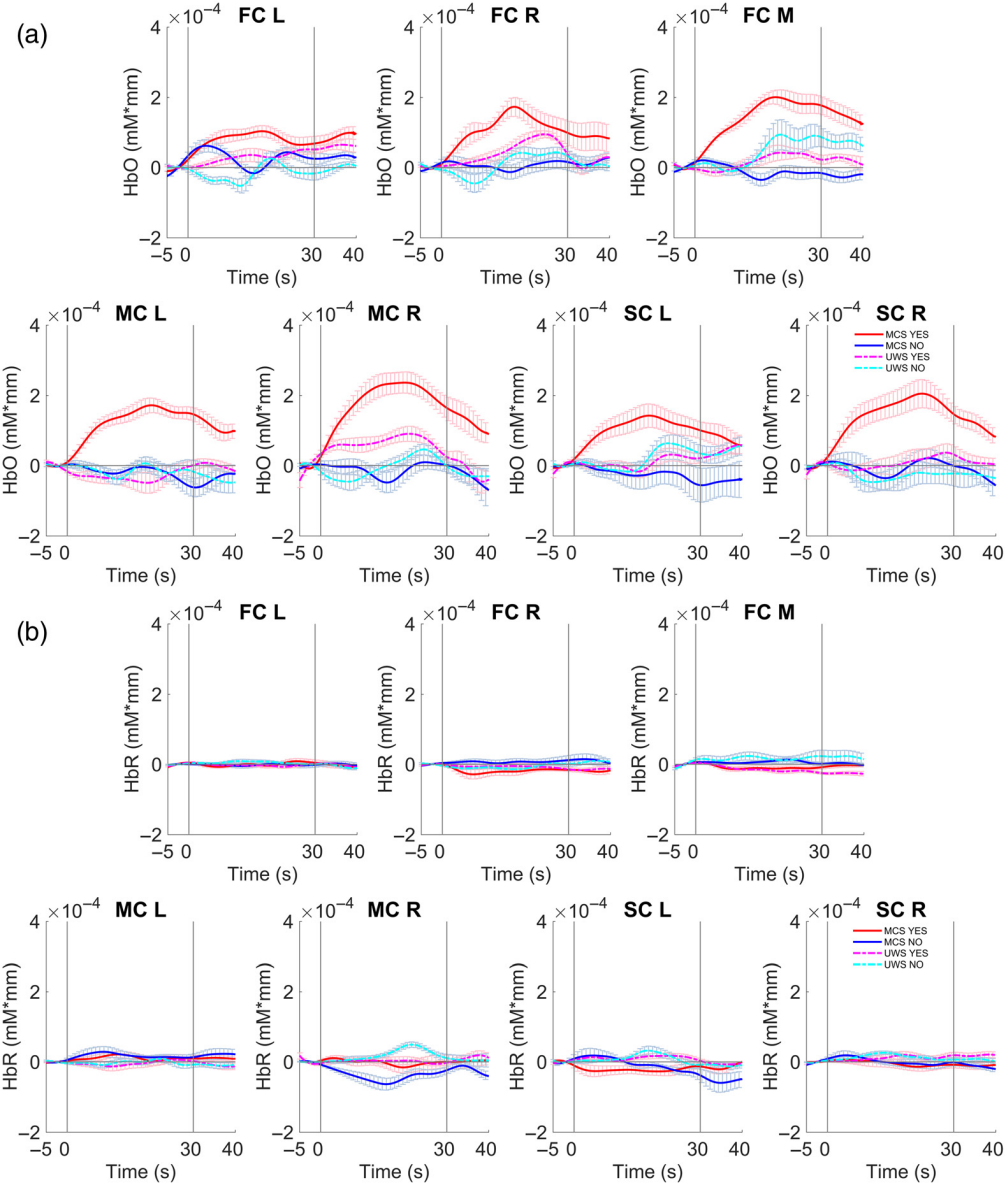
Time courses of the group-averaged (a) HbO and (b) HbR concentrations for the MCS and UWS groups during the command-driven MI task. The red and blue lines indicate the hemodynamic responses for the “YES” and “NO” questions for the MCS, respectively. The dashed magenta and cyan lines indicate the hemodynamic responses for the “YES” and “NO” questions for the UWS, respectively. The duration of the response period is indicated by the space between the two gray lines. The shadowed areas indicate the SEs of the mean data.

**Fig. 5:**
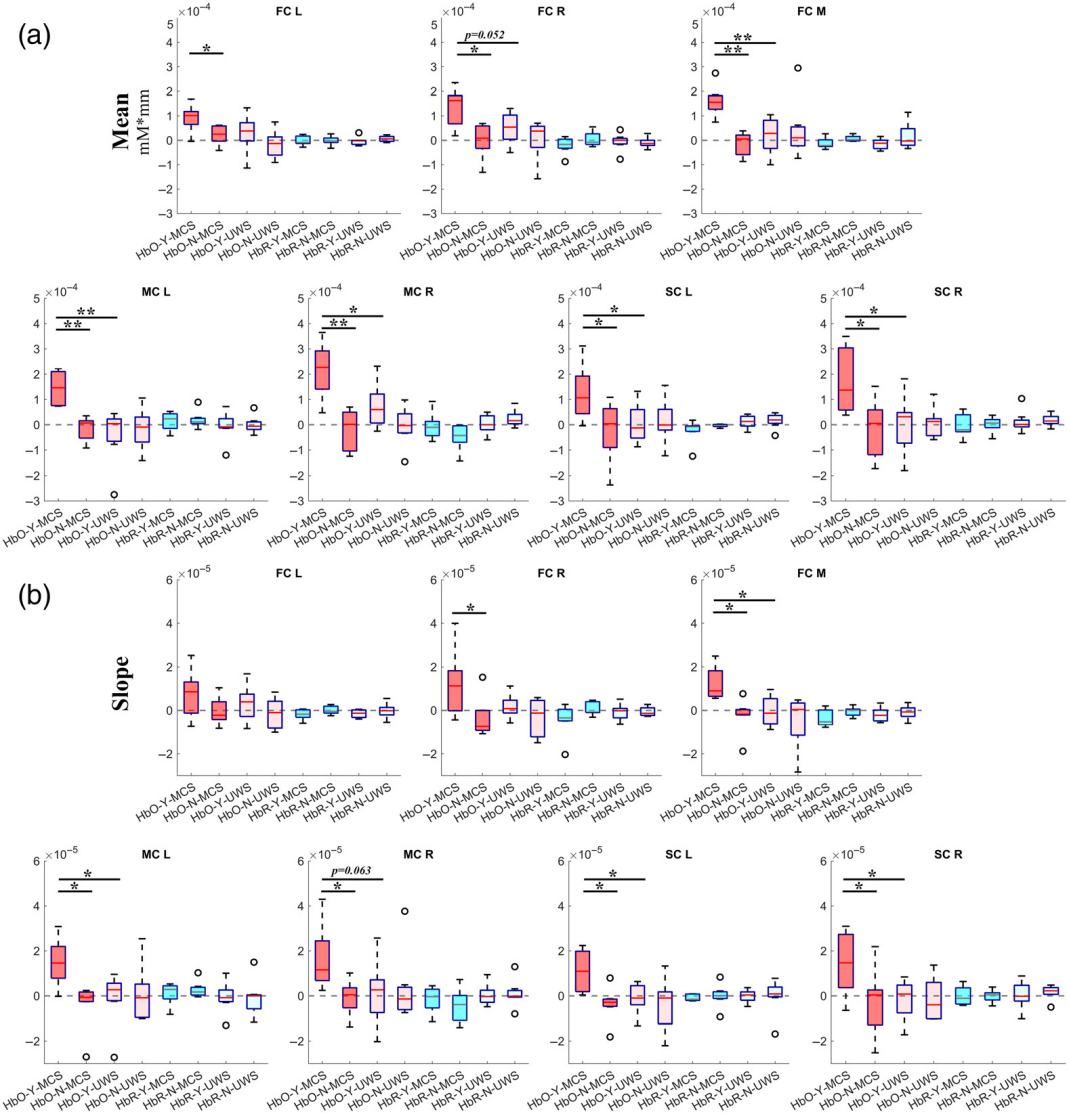
Comparison of the changes in the (a) mean and (b) slope of the hemodynamic responses between the MCS and UWS groups for the command-driven MI tasks. Error bars indicate the SE. MCS, minimally conscious state; UWS, unresponsive wakefulness syndrome; HbO, oxygenated hemoglobin; and HbR, deoxygenated hemoglobin. Y, the “YES” question and N, the “NO” question. *, p<0.05 and **, p<0.01.

The time courses of the group-averaged hemodynamic responses for the MCS and UWS groups during the command-driven MI tasks are illustrated in Fig. 4. The quantitative comparisons of the changes in mean and slope values of the hemodynamic responses are shown in Fig. 5.

As shown in Fig. 4, the distributions of the group-averaged hemodynamic responses were obviously different between the MCS and UWS groups in several brain areas. Specifically, for the patients with MCS, compared to the relatively stable baseline period, the group-averaged HbO concentrations for the “YES” questions over the PFC, primary MC, and primary SC consistently increased to a higher level after the onset of the MI task. When the MI task period was over, the HbO concentrations for the “YES” questions gradually returned to the baseline. The distribution patterns of the HbO concentrations for the “YES” questions for the MCS groups were similar to the “typical” hemodynamic responses, which were characterized by increases in HbO concentration companied by decreases in HbR concentration. However, the distributions of the HbO concentrations for the “NO” questions were quite different from those for the “YES” questions. Specifically, the HbO concentrations for the “NO” questions over the prefrontal, primary motor, and primary sensorimotor areas fluctuated slightly at the baseline level, but the changes did not reach significance. However, there were no significant oscillations in HbR concentrations for either the “YES” or the “NO” questions throughout the task.

Quantitatively, as shown in Fig. 5, the activated mean values of the HbO concentration for the “YES” questions were consistently larger than those for the “NO” questions over the left side of the primary MC (p=0.001 for MC_L) and primary SC (p=0.037 for SC_L) at the group level. Moreover, the activated mean values of the HbO concentration for the “YES” questions over the right side of the primary MC (p=0.002 for MC_R) and primary SC (p=0.023 for SC_R) were significantly larger than those for the “NO” questions. Additionally, the changes in mean values of the HbO concentration for the “YES” questions were significantly larger than those for the “NO” questions for the left side (p=0.024 for FC_L), the right side (p=0.011 for FC_R), and the medial area (p=0.001 for FC_M) of the frontal cortex. For the MCS group, the slope values of the HbO concentration for the “YES” questions were consistently larger than those for the “NO” questions over the left side of the primary MC (p=0.017 for MC_L) and primary SC (p=0.018 for SC_L). The slope values of the HbO concentration for the “YES” questions over the right side of the primary MC (p=0.022 for MC_R) and primary SC (p=0.046 for SC_R) were obviously larger than those for the “NO” questions. Moreover, the slope values of the HbO concentration for the “YES” questions were larger than those for the “NO” questions over the right side (p=0.045 for FC_R) and medial areas (p=0.018 for FC_M) of the frontal cortex. However, for the HbR measurement, the changes of the HbR concentrations were relatively small and no significant differences were found between the “YES” questions and the “NO” questions across the different brain areas.

Conversely, for the patients with UWS, the hemodynamic responses of the MI tasks were fluctuated at relatively low amplitudes around the baseline level and the changes were not statistically significant. Specifically, during the baseline period, the hemodynamic responses over the prefrontal, motor, and sensorimotor areas were relatively stable. After the onset of the MI task, the HbO concentrations for the “YES” and “NO” questions over the primary MC, the primary SC, and the PFC fluctuated at relatively low amplitudes during the response period, but the changes were not significant. Similarly, there were no significant changes in HbR concentrations throughout the MI task. Quantitatively, for the patients with UWS, no significant differences were found in the mean and slope values of the HbO concentration between the “YES” questions and the “NO” questions over the prefrontal, primary motor, and primary SCs.

To investigate the differences in brain functional activation, the task-evoked hemodynamic responses of the MCS and UWS groups were further compared quantitatively. Specifically, the activated mean values of the HbO concentration of the MCS group under the “YES” questions were significantly larger than those of the UWS group over the primary MC (p=0.006 for MC_L, p=0.032 for MC_R). The differences in the mean values of the HbO concentration over the primary SC between the two groups were statistically significant (p=0.039 for SC_L, p=0.033 for SC_R). Note that the mean values of the HbO concentration of the MCS group were obviously larger than those of the UWS group over the PFC (p=0.052 for FC_R, p=0.006 for FC_M). Additionally, the differences in the slope of the HbO concentration between the MCS and UWS groups under the “YES” questions were larger than those of the UWS group over the primary MC (p=0.032 for MC_L, p=0.063 for MC_R) and primary SC (p=0.030 for SC_L, p=0.034 for SC_R). Moreover, the slope values of the HbO concentration of the MCS group of the “YES” questions were significantly larger than those of the UWS group over the medial area of the PFC (p=0.025 for FC_M).

### 3.2 Correlation Between the Task-Evoked Hemodynamic Responses and the CRS-R Scores

**Fig. 6:**
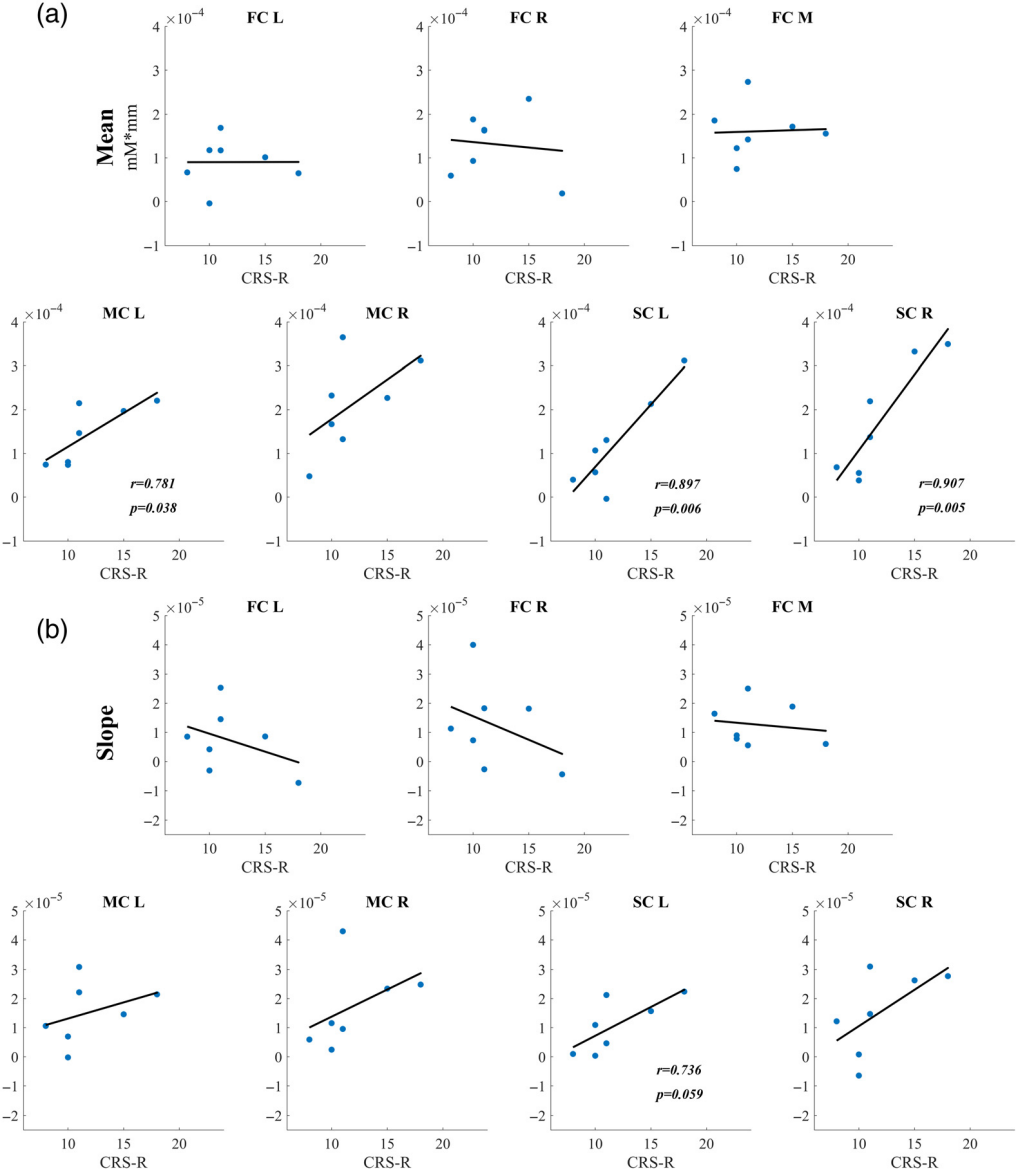
Correlation results between the (a) mean and (b) slope of the hemodynamic responses and the CRS-R (t1) scores for the patients with MCS.

As shown in Fig. 6, the Pearson correlations between the task-evoked hemodynamic responses and the corresponding CRS-R (t1, the first stage before the fNIRS recordings) scores were calculated separately for the MCS and UWS groups. Specifically, for the MCS group, the correlation values between the mean values of the HbO concentration and CRS-R scores were 0.781 (p=0.038 for MC_L), 0.897 (p=0.006 for SC_L), and 0.907 (p=0.005 for SC_R). The correlation result between the slope value and the CRS-R (t1) score was 0.736 (p=0.059 for SC_L). However, for the patients with UWS, the correlation values between the hemodynamic responses and the CRS-R (t1) scores did not reach statistical significance.

Additionally, to further investigate the relationship between the hemodynamic responses and the clinical behavioral evaluation, the correlation values between the characteristics of hemodynamic responses and the CRS-R (t2, the second stage which occurred 6 months after the fNIRS recordings) were also calculated quantitatively. However, no significant results were found for the MCS and the UWS groups.

### 3.3 Comparison of the Subscores of CRS-R Between Different Stages for Patients with DOC

**Fig. 7:**
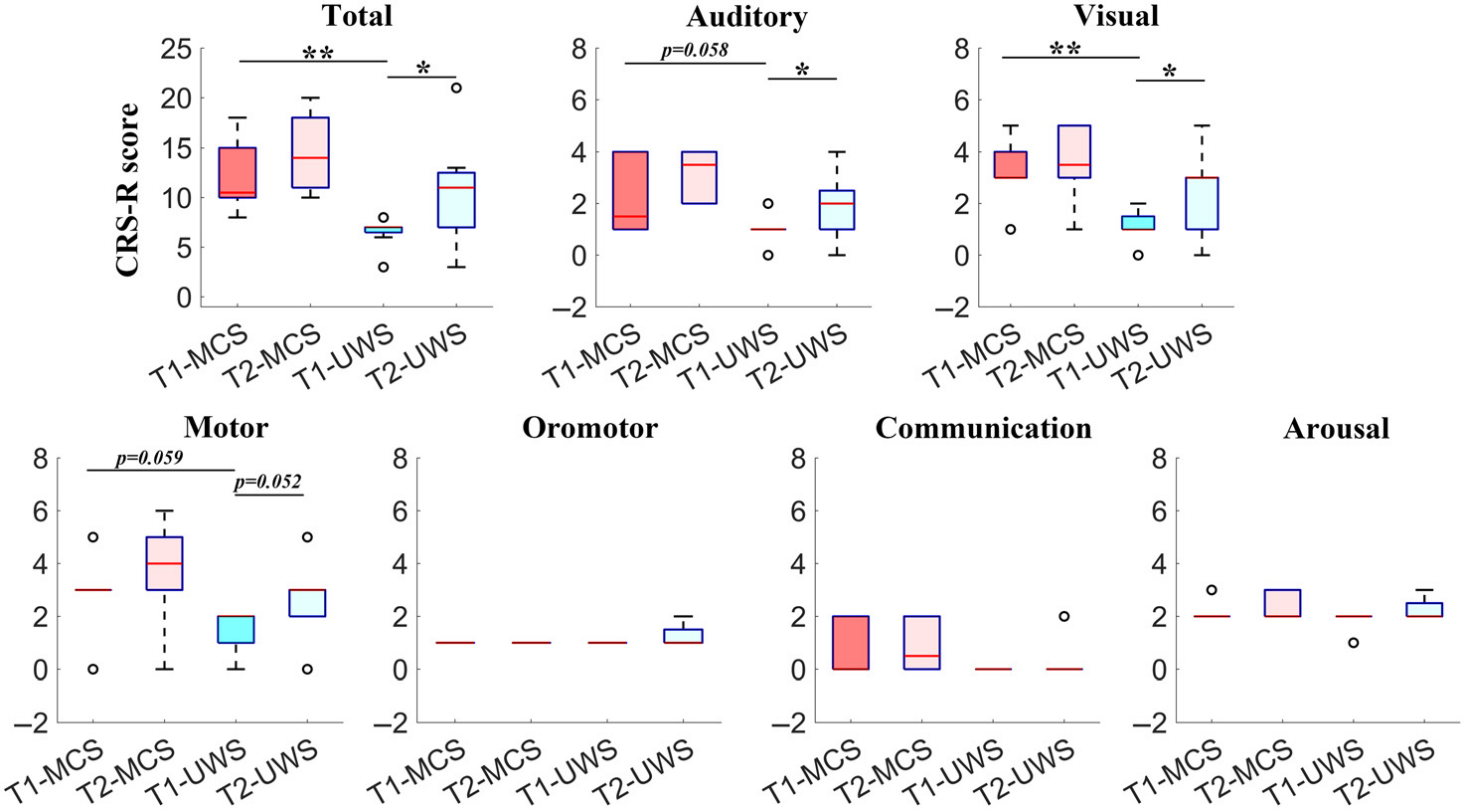
Comparison of the CRS-R scores at the different stages for the MCS and UWS groups. T1 indicates the time for CRS-R (t1) assement before the fNIRS acquisition. T2 indicates the time for CRS-R (t2) assement 6 months after the fNIRS acquisition. *, p<0.05 and **, p<0.01.

The comparisons of the subscores of the CRS-R between the different stages for the MCS and UWS groups are shown in Fig. 7. Paired t-tests were conducted to compare the difference in CRS-R scores in the patients with DOC. The results showed that the CRS-R scores of the UWS group improved significantly in both the total scores and the subscores. Specifically, the total scores of the CRS-R of the UWS group improved significantly (p=0.043). The subscores in the auditory (p=0.021), visual (p=0.038), and motor (p=0.052) subtests also obviously increased after 6 months. However, no significant improvement in the oromotor, communication, or arousal abilities were found for the patients with UWS. For the patients with MCS, overall, the results showed no significant differences in the total and sub-scores of the CRS-R between the two behavioral evaluations. To further investigate the differences between the MCS and UWS groups, the total and subscores of the CRS-R were compared between two groups. Specifically, the results showed that the total score of CRS-R of the MCS group was significantly larger than that of the UWS group (p=0.008). The subscores in the auditory (p=0.058), visual (p=0.007), and motor (p=0.059) subtests of the MCS group were larger than those of the UWS group. However, no significant difference in the oromotor, communication, or arousal abilities was found between the MCS and UWS groups.

## 4 Discussion and Conclusions

Evaluation of the command-following ability of the patients with DOC is a central component of the bedside clinical assessment and diagnostic impression. In the current study, fNIRS was used to measure changes in the hemodynamics of patients with DOC during the command-driven MI tasks. The characteristics of the hemodynamic responses were compared to investigate the spatiotemporal differences between the MCS and UWS groups. In addition, to explore the relationship between objective hemodynamic features and clinical behavioral evaluation, the correlations between the hemodynamic responses and the CRS-R scores were also studied.

### 4.1 Differences in the Hemodynamic Responses Between the MCS and UWS Groups

In this study, the differences between the MCS and UWS groups in functional activation and residual awareness were investigated using fNIRS-based command-driven MI tasks utilizing “YES” and “NO” questions. The complex etiologies and neurobiological mechanisms underlying DOC, along with individual variabilities and fluctuations in arousal between different patients may have contributed to differences in the subject-specific hemodynamic responses. Despite intersubject differences, several pieces of valuable information were found in this study at the group level.

As shown in Fig 4, the distribution patterns of the dynamic fluctuations in hemodynamic responses differed between the MCS and UWS groups. Significant differences in the spatiotemporal distribution of the hemodynamic responses were found between the MCS and UWS groups. Specifically, at the group level, the patients with MCS showed stronger and more rapid changes in HbO concentrations in response to the “YES” questions than to the “NO” questions (Figs. 4 and 5), indicating that the MCS patients exhibited hemodynamic responses that were highly similar to those of people without brain impairments.^22^^,^^26^ Regional brain activation is associated with local increases in cerebral blood supply that exceed the corresponding metabolic demand. The result of this would be that increases in HbO and HbT measurements accompanied by a decrease in HbR measurement can be expected to be recorded in target brain areas using fNIRS system.^38^ The group pattern of change in the hemodynamic responses that we found for the MCS patients are in line with those found in previous studies.^17^^,^^19^ In addition, the hemodynamic responses evoked by the command-driven MI task showed significantly larger amplitudes in the patients with MCS than in the patients with UWS. As shown in Fig. 4, at the group level, the task-evoked hemodynamic responses of the “YES” questions of the MCS group were similar to the typical hemodynamic responses of normal individuals, in that they consisted of a rapid increase in HbO concentration and a corresponding slower, smaller amplitude decrease in HbR concentration.^26^^,^^39^ It is likely that HbO measurement is more sensitive and has a higher SNR and greater robustness to cross-talk than HbR measurement.^32^^,^^39^^,^^40^ It has been reported that the retest reliability and stability of HbO measurement are larger than those of HbR measurement.^41^

Quantitatively, the mean and slope values of the hemodynamic responses during the command-driven MI tasks were extracted to investigate the differences within and between the two groups. For the patients with MCS, the “YES” questions showed significantly higher HbO concentrations than the “NO” questions over the motor-related areas. Specifically, significant differences between the “YES” questions and the “NO” questions in terms of the mean and slope values of the HbO concentrations were found over the primary MC, primary SC, and prefrontal areas for the MCS group. In contrast, there were no significant changes in the HbR concentrations for the MCS group. These results suggest that the task synchronous hemodynamic response patterns of the MCS group might be recognized as an indication of active brain activation and therefore residual awareness. However, the UWS group did not show any significant differences in the hemodynamic responses for either the “YES” or “NO” questions at the group level. Importantly, the mean and slope values of the hemodynamic responses evoked by the command-driven MI task were significantly larger in the MCS group than those of the UWS group over the motor-related brain areas as well as the PFC. This findings differs from the previous study,^17^ which reported that no significant differences in hemodynamic responses were found between the MCS and UWS groups. A review^42^ showed that the statistically significant differences between the MCS and UWS patients were found in approximately half of studies. Such differences could be due in part to the differences in etiology of patients with DOC, experimental design, data analysis, etc. Further exploratory studies need to be done to investigate the differences between the two groups.

Interestingly, the bilateral PFC and bilateral motor-related cortex were activated by the MI task in the MCS group in this study. Several previous studies also reported stronger activation in the bilateral PFC and motor-related cortex during a MI task.^39^^,^^43^ In the past few years, accumulating evidence has indicated that MI and the corresponding movement execution largely share similar motor-related brain circuits, including the primary motor, supplementary motor, premotor, primary SC, and prefrontal cortices, although the evoked activation during MI is lower than during motor execution.^39^^,^^44^ The PFC is responsible for the higher-level information processing (e.g., judgment, motor planning, motor control, etc.) that is required for motor behavior.^39^ Therefore, one likely reason why the task-evoked activation was found over the bilateral PFC is that command-driven MI tasks may require more focused attention for the patients to complete. The current findings further confirmed the importance of the PFC in motor-related activation in patients with DOC. In addition, the group-averaged hemodynamic responses evoked by the command-driven MI task showed no significant differences between the left and right hemispheres. Our results were in line with the previous studies.^17^^,^^19^^,^^22^ Motor execution investigations showed that evoked hemodynamic responses are more pronounced over the contralateral hemisphere, but MI investigations often showed bilateral evoked hemodynamic responses.^43^^,^^45^ Another more likely reason could be that playing badminton often involves whole body movements; therefore, bilateral activations were found at the group level.

### 4.2 Correlation Between the Hemodynamic Responses and the CRS-R Scores

To explore the underlying relationships between the patients’ behavioral presentation and the hemodynamic activations, Pearson correlations were calculated between the CRS-R scores and the mean/slope values of the hemodynamic responses. Specifically, for the patients with MCS, the quantitative comparison showed significant correlations between the hemodynamic responses and the CRS-R (t1, the first stage before the fNIRS recordings) scores. However, for the patients with UWS, the results did not show any statistical correlation between the CRS-R (t1) scores and the mean/slope values of the hemodynamic responses. It was reported that higher CRS-R scores often indicate higher level of consciousness and less brain damage,^24^^,^^46^ thus causing patients to exhibit larger activations during MI tasks. fNIRS features (especially the mean values of the hemodynamic responses) are highly indicative of the residual cognitive ability of patients with DOC. The strong correlations between the fNIRS parameters and the CRS-R (t1) scores in the MCS patients are significant clinically, indicating that fNIRS is feasible for evaluating residual awareness in patients with DOC. These findings show a way to meet the 2020 European Academy of Neurology guidelines^12^ that behavioral assessments should be combined with multimodal neuroimaging technologies to establish a more accurate and robust evaluation of residual awareness in this challenging patient population.

In addition, the ability of fNIRS-based hemodynamic features as a potential prognostic tool for patients with DOC was further evaluated in this study. Specifically, the correlation between the hemodynamic responses and the CRS-R (t2, the second stage, which occurred six months after the fNIRS recordings) was calculated quantitatively. However, the correlation values between the hemodynamic responses and the CRS-R (t2) scores were not statistically significant for both the MCS and UWS groups. One of the possible reasons is the relatively small sample size with heterogeneous etiology. Another possible reason may be due to the different neurorehabilitation therapy for these patients. The current findings suggested that since the fNIRS-based hemodynamic features were not significantly correlated with the CRS-R (t2) scores, the fNIRS-based hemodynamic features, at least in this study, could not be used as prognostic markers. Nonetheless, whether the fNIRS-based hemodynamic characteristics can be used as potential markers for prognosis of patients with DOC should be further testified. In fact, there’s still a lot work to be done in clinical practice, such as: the accumulation of large samples with multiple centers, the optimization of clinical rehabilitation schemes, and the improvement of experimental designs and data processing methods.

### 4.3 Application of fNIRS-Based Active Paradigm in Disorders of Consciousness

In clinical environments, using active command paradigm to detect residual awareness in patients with DOC is essential, because they can identify unequivocal signs of awareness, such as command-following responses and cognitive-emotional responses to external stimulations.^47^ The task-based active paradigms that require subjects to modulate their brain activation in response to external stimuli, provide objective biomarker that helps distinguish between MCS and UWS groups. Compared to a passive paradigm, as well as with a resting state paradigm, such an active paradigm is similar to a clinical behavioral examination, but it may credibly detect a higher level of residual awareness in patients with DOC. Several studies based on such an active paradigm found that although some patients with DOC who appear to be unresponsive are able to intentionally modulate their brain activity in accordance with external commands and even occasionally communicate with the outside world by answering yes/no questions by MI tasks.^9^^,^^10^^,^^12^^,^^48^ The MI is a dynamic mental process during which a skilled movement is mentally simulated without any overt motor output, has been widely used in the fields of sports science and BCI and has shown promise as a rehabilitative treatment for patients with brain injury, such as Parkinson’s disease, stroke, etc.^49^^–^^52^ The MI is a promising active paradigm that can relatively easily be used to detect residual awareness in patients with DOC.

Therefore, in this study, the fNIRS-based active command-driven MI tasks were used to detect the residual awareness in patients with DOC and to explore the spatiotemporal differences between the MCS and UWS groups. In this study, five patients with MCS exhibited command-following hemodynamic responses to command-driven MI tasks in more than one “YES” question. For instance, patient No. 12 was a 49-year-old woman and has been MCS for more than 12 years. She had the ability of moving her upper extremities in response to commands and showing simple functional interactive communication with others, but not consistently. Patient No. 9 was a 33-year-old man, he sometimes responded to commands such as open and close his eyes and showed simple functional communication with others, albeit inconsistently. Patient No. 2 was a 35-year-old man, he regained the ability to pursue visual cues, simple functional communication, and functional use of different objects after 6-month recovery. Note that one patient (patient No. 7) with UWS also demonstrated command-following hemodynamic responses to the MI tasks, indicating covert awareness. Interestingly, during the follow-up visit process, the CRS-R score of this patient improved from 7 (211102) to 21 (455223). This patient has recovered well and has, as of this writing, regained the ability to pursue visual cues, use objects, and even communicate with others. Other studies have reported that ∼15% of patients who appear to be unresponsive may have a CMD.^12^^,^^53^ The current study further confirmed the feasibility and availability of fNIRS-based command-driven active paradigm in detecting residual awareness in patients with DOC. More importantly, this study fully investigated the spatiotemporal characteristic differences in hemodynamic responses between the MCS and UWS groups based on fNIRS technique. It also provided more evidence of the potential for fNIRS-based BCIs to aid in basic communication and to improve the diagnostic accuracy for patients who appear to be behaviorally nonresponsive.

### 4.4 Limitations of the Current Study

One of the limitations of this study is the varied causes of brain injuries among the patients with DOC. In further explorative studies, the patients’ enrollment should be more carefully controlled and inter-subject variability in etiology and damaged brain areas should be taken into account. A second limitation is that there was only one type of MI task (badminton playing). However, it has been reported that hemodynamic responses may be modulated by the intensity, frequency, and complexity of the task.^7^^,^^54^ Therefore, in future studies, MI tasks that vary in complexity (such as hand squeezing, finger tapping, imagining badminton playing, etc.) should be used to fully evaluate the consciousness in patients with DOC. Because of the exploratory nature of this study and the limited sample size, the current findings should be further investigated and validated in larger samples as well as using advanced experimental paradigms, improved processing methods, and comprehensive behavioral assessments.

Despite these limitations, this study provided further evidence for the ability of fNIRS to detect the residual awareness of patients with DOC. These results could benefit future BCI studies and further provide guidance and support for the clinical diagnosis, therapy, and prognosis of patients with DOC. In addition, the command-following active experimental protocol used in this study could provide useful insights into identifying more accurate and reliable evaluations of consciousness and investigating higher levels of functional activation for this challenging patient population.

## Code, Data, and Materials Availability

The datasets generated and/or analyzed during the current study are available from the corresponding author on reasonable request.
